# Amyotrophic Lateral Sclerosis-Related Respiratory Failure and Association With Inappropriate Secretion Syndrome of the Antidiuretic Hormone

**DOI:** 10.7759/cureus.34851

**Published:** 2023-02-10

**Authors:** Tiago Ceriz, Andreia Diegues, Sérgio R Alves, Pedro Simões, Miriam P Blanco

**Affiliations:** 1 Internal Medicine Department, Unidade Local de Saúde do Nordeste, Bragança, PRT; 2 Internal Medicine Department, Unidade Local de Saúde de Bragança, Bragança, PRT

**Keywords:** narcosis, respiratory failure, amyotrophic lateral sclerosis, hyponatremia, inappropriate secretion of antidiuretic hormone

## Abstract

There is an unclear association between the syndrome of inappropriate antidiuretic hormone secretion (SIADH) and amyotrophic lateral sclerosis (ALS), with few reports in the literature. We report the case of an 80-year-old man admitted to our emergency room with asthenia, dysphonia, dysphagia, weight loss, and euvolemic hyponatremia, indicating a SIADH. Posteriorly, the patient also developed respiratory failure, which, in association with the previous clinical presentation, led to the diagnosis of ALS. During her permanence at the hospital, the hyponatremia improved with noninvasive positive-pressure ventilation, and the association between these two identities was made. This case also shows that patients with ALS commonly suffer from chronic respiratory failure and still have a reserved prognosis.

## Introduction

Hyponatremia is the most common hydroelectrolytic disorder in hospitalized patients. It is defined as excess water in relation to sodium in the extracellular fluid [1]. There are different etiologies, with the syndrome of inappropriate antidiuretic hormone secretion (SIADH) being one of the more frequent causes [2]. SIADH is a disorder of impaired water excretion caused by the inability to suppress the secretion of antidiuretic hormone (ADH) [3]. This should be suspected in any patient with hyponatremia, hypoosmolality, and urine osmolality above 100 mosmol/kg [3]. The etiology includes pathologies from different categories, such as drugs, neoplasia, disorders of the central nervous system (CNS), pulmonary diseases, surgery, hormone deficiency, HIV infection, and idiopathic causes. A large group of CNS disorders can be associated with SIADH, but the underlying mechanism that leads to hyponatremia in this particular group remains unclear [4]. However, a few hypotheses are that in some cases, CNS disorders may stimulate the release of antidiuretic hormone from the neurohypophysis, and in other cases, severe restrictive respiratory failure requiring mechanical ventilation may cause hypersecretion of antidiuretic hormone, as in amyotrophic lateral sclerosis (ALS). Although rare, the association between SIADH and ALS has been described in a few cases. ALS is a debilitating, progressive disease with degeneration of motor neurons in the brain and spinal cord, causing weakness, muscle atrophy, fasciculations, and spasticity [5], as described in Canadian best practice recommendations for the management of amyotrophic lateral sclerosis [5]. It is an etiologically diverse clinical entity that is the result of a combination of multiple preceding aberrations. The most common presentation, in about 70% of patients, is symptom onset in the limbs, with extremity weakness and impairment in mobility [5]. Bulbar onset, with oropharyngeal muscle involvement affecting swallowing and speech, occurs in about 25% of cases [5]. In addition to motor impairment, degeneration in the frontal and temporal lobes, resulting in cognitive or behavioral impairments, occurs in up to 50% of patients [5]. The diagnosis of ALS is primarily clinical, but electrodiagnostic studies can further support the diagnosis, especially if the clinical picture is unclear. There are four categories that reflect the degree of clinical involvement described by the World Federation of Neurology (WFN) [5].

Like the Canadian best practice recommendations for the management of amyotrophic lateral sclerosis, over time, strength progressively declines, and patients typically die from respiratory failure within five years of diagnosis. Despite increased research efforts in recent years, treatment options for ALS remain limited, and patient care is focused primarily on managing symptoms and optimizing function and quality of life [5], provided by a multidisciplinary team [6]. The treatment of hyponatremia in SIADH includes the treatment of the underlying cause, initial therapy to raise the serum sodium, and sometimes prolonged therapy when persistent SIADH is identified [1,6]. Here, we describe a case of a patient who initially presented with hyponatremia of multifactorial etiology and maintained the imbalance after the removal of all the predisposing factors and drugs. Additionally, besides hyponatremia, the patient also had unspecified dysphagia that evolved into narcosis, which led to the diagnosis of ALS [7]. These two factors confirmed the diagnosis of ALS associated with SIADH.

## Case presentation

An 80-year-old man was admitted to the emergency department due to asthenia, dysphonia, intermittent dysphagia for liquids and solids, and a weight loss of 14 kg in four months. This patient had prostatic benign hyperplasia, type 2 diabetes mellitus, atrial fibrillation, ischemic heart disease, and chronic heart failure as comorbidities and was being treated with furosemide, metolazone, eplerenone, carvedilol, amiodarone, apixaban, tamsulosin, omeprazole, ticagrelor, and acetylsalicylic acid. At admission, the examination revealed an arterial blood pressure of 108/59 mmHg, a heart rate of 60 bpm, and oxygen saturation levels of 95%. Of note is that the patient was euvolemic and had significant weight loss. A neurological examination revealed muscular atrophy without further alterations. The initial laboratory study showed hyponatremia (sodium levels of 122 mg/dL) and serum hypoosmolarity (253 mOsm/kg, with normal values between 280 and 300 mOsm/kg). Five days after thiazide suspension, sodium levels were 126 mg/dL and serum osmolarity was 264 mOsm/kg, with increased urinary sodium excretion, urinary osmolarity of 520 mOsm/kg, fractional sodium excretion of 1.5%, and without clinical signs of volume depletion or overload of extracellular fluid, suggesting SIADH (Tables 1-2).

**Table 1:** Blood laboratory results - hospital admission ALP: alkaline phosphatase; ALT: alanine aminotransferase; AST: aspartate aminotransferase; GGT: gamma-glutamyl transferase; LDH: lactate dehydrogenase.

Parameter	Result		Reference range
	Admission	After stop thiazide	
Blood tests			
Hemoglobin	14.1	-	14-17.5 (g/dL)
Leukocytosis	5.6	-	4.4–11.3 (×10^9^/L)
Platelet	223	-	150–450 (×10^9^/L)
Sodium	122	126	134–145 (mEq/L)
Potassium	3.6	3.8	3.5–5.1 (mEq/L)
Chloride	101	98	98–107 (mEq/L)
Urea	20	19	17–43 (mg/dL)
Creatinine	1.35	1.28	0.91–1.44 (mg/dL)
Glucose	95	93	74–106 (mg/dL)
AST	38	-	<45 (IU/L)
ALT	31	-	<35 (IU/L)
ALP	110	-	30–120 (IU/L)
GGT	42	-	<55 (IU/L)
Total bilirubin	1.1	-	0.3–1.2 (mg/dL)
Serum albumin	3.7	-	3.4–4.8 (g/dL)
Total protein	7.3	-	6.6–8.3 (g/dL)
Creatine kinase	178	157	<171 (IU/L)
LDH	237	222	<248 (U/L)
Reactive protein	0.05	-	<0.1 (mg/dL)

**Table 2:** Urinalysis results - hospital admission

Parameter	Result	Reference range
Urinalysis		
Urine osmolarity	520	300–900 (mOsm/kg)
Urine sodium	88	40–220 (mEq/L)
Urine potassium	31	20–60 (mEq/L)
Urine creatinine	64	22–328 (mg/dL)

The chest X-ray at admission showed no acute pleuroparenchymal abnormalities. Other causes of hyponatremia were excluded during the hospital stay, namely thyroid dysfunction, renal failure, and glucocorticoid deficit (Table 3).

**Table 3:** Laboratory results - study during hospitalization ACTH: adrenocorticotropic hormone; BNP: B-type natriuretic peptide; C: complement; CMV: cytomegalovirus; HDL: high-density lipoprotein; Ig: immunoglobulin; LDL: low-density lipoprotein; TSH: thyroid stimulating hormone; T4: thyroxine; VDRL: venereal disease research laboratory.

Parameter	Result	Reference range
BNP	32	<100 (pg/mL)
B12 vitamin	435	187–883 (pg/mL)
Acid folic	12	3.1–20.5 (ng/mL)
Ferrum	88	60–180 (ug/dL)
Ferritin	200	4.63–204.00 (ng/mL)
Transferrin saturation	26	20–50 (%)
Uric acid	3	2.6–6.0 (mg/dL)
Total cholesterol	180	<200 (mg/dL)
LDL cholesterol	78	<155 (mg/dL)
HDL cholesterol	47	30–60 (mg/dL)
Triglycerides	143	<150 (mg/dL)
TSH	0.77	0.35–4.94 (uUI/ml)
Free T4	1.51	0.70–1.48 (ng/dL)
Morning serum cortisol	15.1	6.2–19.4 (ug/dL)
ACTH	29.2	7.2–63.3 (pg/mL)
VDRL	Negative	
HIV 1+2	Negative	
Antibodies anti-CMV IgG	1647.3	<6.0 - negative (UA/mL)
Antibodies anti-CMV IgM	0,08	<0.85 index- negative >1.00 index - positive
HBsAg	0.19	<1 - negative
Anti-HBs	>1000.00	<10.00 - no reactive
Anti-HBc	0.88	<1.000 - no reactive
Anti-HCV	0.11	<1 - negative
IgG	13.15	7.0–16 (g/L)
IgA	2.7	0.70–4.00 (g/L)
IgM	1.01	0.4–2.3 (g/L)
C3	1.00	0.9–1.8 (g/L)
C4	0.33	0.1–0.4 (g/L)

**Figure 1:**
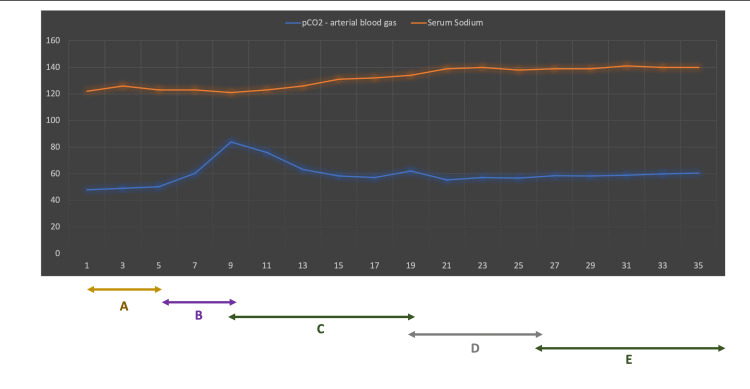
Clinical course, laboratory results and treatment evolution during hospitalization Serum sodium (mmol/L) and pCO_2_ (mmHg) arterial blood gas evolution during hospitalization and clinical course and treatment description as below. A - First five days: Patient stop diuretics and started NaCl infusion. B - At 5th day new sodium worsening despite treatment. Clinically euvolemia, SIADH suspicion. Patient started fluid restriction with slightly improvement. C - At 9th day observed hypercapnia coma, starting NPPV with improvement of both CO_2_ and Na^+ ^levels. D - At 19th day patient developing respiratory failure with severe hypoxemia and slightly worsening hypercapnia, needing invasive ventilation. E - At 27th day extubation to NPPV. For motor neuron disease suspicion at 31th day riluzole was started. Although all effort patient day at 48th.

The etiological investigation and treatment of SIADH became a challenge, with slight correction of hyponatremia initially with the suspension of metolazone but without normalization. A thoracic and abdominopelvic computed tomography (CT) was performed, revealing calcified lymphadenopathy in the right hilum and subcarinal region, no pleural effusion, and no abdominal or pelvic alterations. Cerebral CT did not reveal alterations suggestive of space-occupying lesions, reporting signs of ischemic leukoencephalopathy and permeable cerebrospinal fluid pathways. Neck CT showed pneumatized sinus cavities without soft tissue thickening of the posterior cavum wall or appreciable deviation of the median raphe, describing infracentimetric lymph nodes in the jugular and posterior cervical chains and bilateral submandibular topography. The thyroid had regular contours, normal volume, and homogeneous enhancement. The upper gastrointestinal endoscopy also showed no alterations. Despite instituted measures, such as NaCl infusion at the beginning and then water restriction, a few days later patient´s hyponatremias worsened and clinical dysphagia is more evident, with progressive respiratory insufficiency (RI) and carbon dioxide narcosis with the need for non-invasive positive-pressure ventilation (NPPV), that resulted in improvements, not only in ventilatory dynamics but also in hyponatremia. However, the patient's clinical condition and RI deteriorated again due to the development of nosocomial pneumonia, requiring invasive ventilation and admission to the intensive care unit (ICU). In the ICU, no microbiological isolations were made, and the patient completed a course of antibiotic therapy with ceftriaxone plus clarithromycin. Regardless of the infectious condition improvement, the respiratory failure was not showing the same clinical response. At this point, motor neuron disease is highly suspected, and the patient started riluzole with a slight improvement in his condition. Electromyography was then performed, revealing criteria for active denervation on the four limbs and trunk-findings that, together with the clinical evolution, are compatible with the diagnosis of ALS. Later, difficulty in ventilatory weaning led to a tracheostomy and a change to hybrid ventilation; however, the patient's clinical condition kept declining in spite of all the measures taken, and the patient died after 48 days of hospitalization (Figure 1).

## Discussion

Initially, the diagnosis of SIADH was not made because of the history of diuretic use, but with the elimination of this factor and the presence of clinical euvolemia, urinary osmolarity exceeding 100 mOsm/kg, and other urinary results, the diagnosis was confirmed [3]. The diagnosis of ALS was made by clinic suspicion and confirmed with electromyography results. In SIADH, the only definitive treatment is the control of its underlying cause [1,3], so it is important to search for it. As described previously, the association between ALS and SIADH is rare, and the majority of cases described refer to the Asian population. The mechanism underlying this association is not fully understood, but intrathoracic circulatory dysfunction due to the atrophy of the respiratory muscles, leading to hypercapnia and hypoxia, may play a role in the development of SIADH, as possibly seen in this case and as described previously by Yoshida et al [2]. First, hypoxia can cause constriction of the pulmonary vessels and decrease the venous return to the left atrium [2]. Furthermore, it can directly or indirectly stimulate the hypothalamus via the carotid baroreceptors, and at the same time, hypercapnia can stimulate ADH hypersecretion via baroreceptors or by inducing acidosis [2]. In this patient´s case, the improvement in sodium level with the use of NPPV corroborates this theory, although there have not been enough studies. There are numerous complications associated with ALS, and in this case, respiratory decline with the necessity of ventilatory support, dysphagia, malnutrition, fatigue, and functional decline due to muscular weakness were observed. Unfortunately, the main treatment available for patients with this condition is symptomatic management and support. As for SIADH, the treatment consists of identification and resolution of the cause; however, in this case, complete management and resolution of the precipitating factors were not possible [1]. Some studies describe Riluzole (50 mg twice per day) as the only drug that has been proven to favorably impact overall survival [2,4,7]. Additional pharmacologic therapies are needed, and several clinical trials are currently in development in efforts to improve a prognosis that is still reserved. There are some factors that, if present at diagnosis, are associated with better survival rates; these include increased weight (mild obesity according to the body mass index), younger age, a higher ALS functional rating scale score, a higher forced vital capacity (FVC), and limb rather than bulbar symptoms. However, none of these factors were present in this patient´s case [5].

## Conclusions

We reported a rare finding of ALS-related respiratory failure that might be associated with SIADH. This case highlights the importance of SIADH investigation and the relevance of early recognition of ALS as a possible cause of SIADH. NPPV may ameliorate the control of SIADH in this clinical setting. So, this shows that supportive therapy for respiratory failure related to ALS improved the Na level in this patient. There are very few case reports supporting this theory in the literature. As was seen in this case, the complications associated with amyotrophic lateral sclerosis were diverse, particularly the respiratory decline and the necessity of further ventilatory support, which when associated with nosocomial infections increased the predisposition to poor prognosis and death.
